# Insight into the Burden of Antimicrobial Resistance among Bacterial Pathogens Isolated from Patients Admitted in ICUs of a Tertiary Care Hospital in India

**DOI:** 10.1155/2024/7403044

**Published:** 2024-01-06

**Authors:** Garima Gautam, Shweta Satija, Ravinder Kaur, Anil Kumar, Divakar Sharma, Megh Singh Dhakad

**Affiliations:** ^1^Indraprastha Apollo Hospital, New Delhi, India; ^2^Department of Microbiology, Lady Hardinge Medical College and Associated Hospitals, New Delhi 110001, India; ^3^Department of Microbiology, Maulana Azad Medical College and Associated Hospitals, New Delhi 110002, India

## Abstract

Intensive care unit (ICU) patients are prone to develop infections by hospital prevalent organisms. The aim of the study was to determine the bacteriological profiles and their drug resistance pattern among different infections in ICU patients of a tertiary care hospital. The record-based retrospective data of culture reports of the patients admitted to all the ICUs of a tertiary care hospital during the period from January 2020 to May 2022 were analyzed. A total of 3,056 samples were obtained from 2308 patients. The infection rate among ICU patients was found to be 53.40%. Isolates belonged equally to males (50.86%) and females (49.14%). The most common culture-positive clinical specimen received was blood (39.08%) followed by respiratory samples (29.45%). *Acinetobacter* sp. (33.02%) was the most common organism isolated from various clinical specimens, followed by *Klebsiella pneumoniae* (20.89%), and *Escherichia coli* (13.8%). More than 80% of *Acinetobacter species* were found to be resistant to third-generation cephalosporins, aminoglycosides, and carbapenems, whereas minocycline (56.31% S) and colistin (100% S) were the most effective drugs. *Klebsiella* sp. was found to be more resistant than *E.coli*, and the least resistance was observed to be tetracycline (43.97%) and doxycycline (55.84%). Among *Staphylococcus aureus*, 82.78% of strains were methicillin-resistant (MRSA). Vancomycin-resistant *Enterococci* (VRE) sp. accounted for 16.67% of the isolates. Evidence-based knowledge regarding the local bacterial organisms and their antimicrobial resistance pattern is pivotal in deciding empirical drug therapy, ultimately leading to the management of antimicrobial resistance (AMR).

## 1. Introduction

Microbial infections and antimicrobial resistance have been recognized as a critical issue worldwide, affecting public health, therefore considering the most important causes of mortality and morbidity [1]. According to INEbase death statistics, in the year 2020, infectious diseases were the third most common cause of death, accounting for 16.4% of the total, which includes identified and suspected COVID-19 cases [2]. Although intensive care units (ICUs) account for fewer than ten percent of total beds, they serve as a factory for the development and spread of microbial infections [3]. In countries where routine infection control measures are implemented extensively, ICUs are still potential sources of nosocomial infections [4]. Accommodation of seriously ill patients who are usually immunocompromised, and undergoing invasive procedures in ICUs, results in a five to seven-fold higher risk of nosocomial infections than other patients [5]. Other factors involved are increased duration of stay, use of immunosuppressive drugs, and prolonged or inappropriate use of broad-spectrum antibiotics [5]. This leads to a huge economic burden on the health system of developing countries. Therefore, we hypothesized to investigate the antimicrobial resistance among bacterial pathogens isolated from patients admitted in ICUs.

The development of antibiotics has been acknowledged as the greatest medical advance in human history. However, antimicrobial resistance (AMR) has been rising due to misuse of these valuable compounds which has ultimately resulted in some infections becoming effectively untreatable [6]. According to a report by the UK government health department, ten million people will die in a year from drug-resistant infections by 2050, if urgent action is not taken. Currently, at least 700,000 people lose their lives each year globally, because of drug resistance in illnesses such as bacterial infections, malaria, HIV/AIDS, or tuberculosis [7]. In addition, the emergence of highly resistant microorganisms in ICUs has become a major threat to patients, leading to worse outcomes and demand for the last line of antimicrobials [8].

Surveillance of AMR is the first and foremost essential step towards curtailing the spread of antimicrobial resistance, forming policies, and for infection prevention and control interventions. Importantly, it is the cornerstone for monitoring the impact of local, national, and global strategies. In 2015, WHO launched the Global Antimicrobial Resistance and Use Surveillance System (GLASS), the first global collaborative effort to standardize AMR surveillance. Similarly, in 2021, the Indian Council of Medical Research (ICMR) also started the Antimicrobial Resistance Surveillance System (i-AMRSS), a promising tool for the collection, management, and analysis of AMR data [9].

AMR surveillance helps to generate baseline data on the pattern of microorganisms in the hospital and their susceptibility profile, which in turn helps in deciding effective and rational empirical treatment. These data vary from country to country, hospital to hospital, and even among different wards of the same hospital. Therefore, the objective of this study was to determine the bacteriological profiles and their drug resistance pattern among different infections in ICU patients of a tertiary care hospital.

## 2. Material and Methods

### 2.1. Settings

This retrospective observational study was carried out in the department of microbiology of a tertiary care hospital in North India from January 2020 to May 2022.

### 2.2. Subjects

The study was undertaken based on reports of bacterial isolates of various clinical specimens from different ICUs, such as medical ICUs (MICUs), surgical ICUs (SICUs), and paediatric ICUs (PICUs), which were submitted to the microbiology laboratory for culture and sensitivity during the study period.

### 2.3. Inclusion Criteria

All the patients who were admitted to various ICUs (medical, surgical, and paediatric ICUs) during the study period and whose reports were retrieved from the laboratory were included in the study. Various sources of clinical specimens included blood, urine, pus, cerebrospinal fluid (CSF), catheter tips, endotracheal tips, drainage fluids (trauma pleural and ascitic), bronchial aspirates (BALs), central venous catheters (CVCs), sputum, and gastric aspirate. Only bacterial isolates were included.

### 2.4. Exclusion Criteria

1. Mixed growths (three or more isolates) per specimen in urine culture only
2. Unsatisfactory sputum samples in accordance with the Bartlett scoring system
3. Any leaked or incorrectly labelled samples
4. Multiple samples of the same type of specimen from a single patient

### 2.5. Ethical Approval

This study was conducted in accordance with the Declaration of Helsinki, and the protocol was approved by the institutional ethics committee (LHMC/IEC/2022/03/78). Consent was taken from study subjects for inclusion in the study.

### 2.6. Methodology

A total of 2308 patients were admitted to the ICUs during the study period, from which 3056 samples were received. All the organisms were identified morphologically and biochemically by a standard laboratory procedure. The received specimens were streaked on the blood agar and MacConkey agar and incubated aerobically overnight at 37°C. Growths isolated from all the samples were identified by observing the colony characteristics and biochemical reactions using standard microbiological methods. Unidentified isolates were subjected to further identification using the VITEK 2 ID system. Antimicrobial susceptibility testing was conducted by the disk diffusion method as per the Clinical and Laboratory Standard Institute (CLSI). The zone of diameter was measured and interpreted as susceptible (S), intermediate (I), and resistant (R) as per the CLSI (2020–22). The control strains used were *E. coli* ATCC 25922, *Pseudomonas aeruginosa* ATCC 27853, and *Staphylococcus aureus* ATCC 29213.

Colistin susceptibility testing was performed using the microbroth dilution test as per CLSI guidelines. The test for extended-spectrum beta-lactamase (ESBL) production was performed using the combined disk diffusion method with antibiotic disks of ceftazidime/clavulanic acid (30/10 mcg) and ceftazidime (30 mcg) (as per CLSI). Cefoxitin disk diffusion (30 *μ*g) was used to detect MRSA. Vancomycin susceptibility testing for *Staphylococcus aureus* was done using vancomycin screen agar method (BHI agar with 6*μ*g/mL of vancomycin).

Statistical analysis: data were analyzed statistically with categorical variables like the proportion of bacterial infections across different ICUs, sample type, age groups, and gender. Patterns of microorganisms, their susceptibility profiles, and sites of infections were also analyzed and expressed as percentages. SPSS software was used for statistical analysis.

## 3. Results

In the present study, a total of 3056 samples were received from 2308 patients admitted to ICUs and were used for data analysis. Of 3056 samples, bacterial pathogens were isolated from 1632 samples (53.40%).

### 3.1. Clinical Specimen and Demographic Profile of Culture-Positive Patients

Of 1632, the majority of isolates were from 1 to 11 years old children (31.21%) followed by adults from 18 to 45 years of age (28.03%). Percentage of culture positivity was highest in PICU (41%), followed by MICUs (30.88%), and SICUs (27.57%). Culture isolates belonged equally to males (50.86%) and females (49.14%). The most common culture-positive clinical specimen received was blood (39.08%), followed by respiratory samples (29.45%), exudates (7.32%), and body fluids (6.71%). The distribution of specimens and demographic details of culture-positive isolates are shown in Table 1.

### 3.2. Distribution of Bacteriological Isolates

*Acinetobacter* sp. (33.02%) was the most common organism isolated from various clinical specimens, followed by *Klebsiella pneumoniae* (20.89%), and *Escherichia coli* (13.8%). Among the Gram-positive organisms, *Staphylococcus aureus* (16.78%) was the most common organism followed by *Enterococcus* sp. (3.73%). Details of the distribution of bacterial isolates are shown in Table 2.

### 3.3. Pattern of Antimicrobial Resistance in Detected Predominant Organisms

The antimicrobial sensitivity pattern of the different major bacterial isolates to different antimicrobials is shown in graphs 1-2. The majority of Gram-negative bacteria (GNB) were resistant to *β*-lactam antimicrobials and *β*-lactam/*β*-lactamase inhibitor combination. High resistance was also shown to quinolone, cotrimoxazole, and to some extent to carbapenem groups.

**Figure 1:**
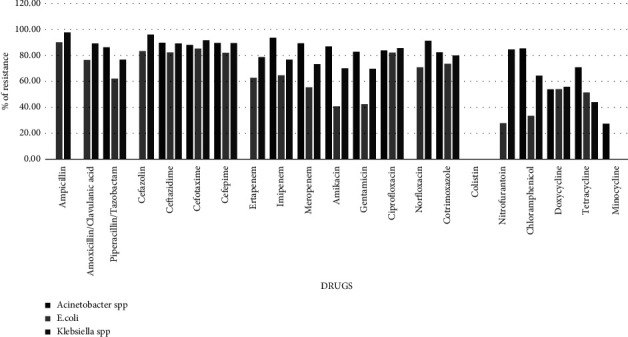
Percentage resistance to various antimicrobials among major Gram-negative bacilli isolated from the ICU patients at LHMC and its associated hospitals.

The susceptibility pattern of *Acinetobacter* spp. showed that almost all the isolates were resistant to all drugs (up to 93% resistant) except doxycycline (53.91% R) and minocycline (27.33% R), as shown in Figure 1. *Escherichia coli (E.coli)* was moderately resistant to tetracycline (51.45%), meropenem (55.5%), piperacillin tazobactam (62.2%), ertapenem (62.8%), and imipenem (64.71%) and least resistant to chloramphenicol (33.3%), amikacin (40.74%), and gentamicin (42.14%). Similarly, *Klebsiella* sp. was found to be least resistant to tetracycline (43.97%) and doxycycline (55.84%) with high resistance varying from 60 to 97% to all other drugs (Figure 1). ESBL production was similar in both the organisms (16%).

**Figure 2:**
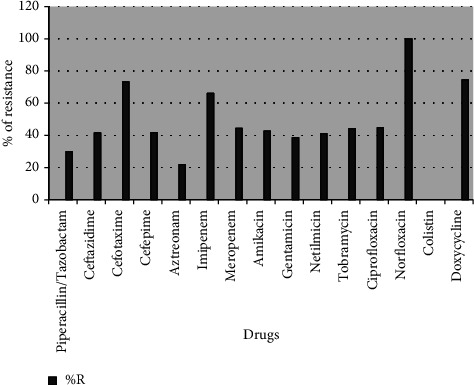
Percentage resistance to various antimicrobials among *Pseudomonas aeruginosa* isolates isolated from the ICU patients at LHMC and its associated hospitals.

Almost half of the isolates of *Pseudomonas aeruginosa* (*P. aeruginosa*) were resistant to *β*-lactam antimicrobials, quinolones, and carbapenems. Least resistance was seen in aztreonam (21.82%) and piperacillin-tazobactam (29.89%) as shown in Figure 2. All the Gram-negative isolates were susceptible to colistin (100% S). Among GNB isolated from urine specimens, *E.coli* was most susceptible to nitrofurantoin followed by *Klebsiella* sp. *Acinetobacter* spp. and *Pseudomonas* spp. were extremely resistant to nitrofurantoin.

*Staphylococcus aureus (S. aureus)* showed the highest percentage of resistance towards penicillin (96.34%) followed by erythromycin (77.01%). On the other hand, linezolid, teicoplanin, and vancomycin displayed absolutely no resistance. Eighty-two percent strains were methicillin-resistant (MRSA). *Enterococci* spp. expressed a high level of resistance to all beta-lactams and norfloxacin (93.75%), erythromycin (81.97%), ciprofloxacin (79.63%), tetracycline (74.58%), and high-level gentamicin (65.57%). Vancomycin-resistant *Enterococci* (VRE) spp. accounted for 16.67% of isolates. No resistance was seen against linezolid (Table 3).

## 4. Discussion

The rapid development and spread of antimicrobial resistance among bacteria are threatening public health worldwide. Multidrug-resistant infections are one of the major causes of mortality and morbidity among patients admitted to hospitals. According to the World Health Organization (WHO), people living in a low-income country are far more likely to die of a communicable disease than of a noncommunicable disease. Despite the global decline, six of the top ten causes of death in low-income countries are communicable diseases [10]. Hence, this study was undertaken to provide insight into the extent of antimicrobial resistance among bacteria isolated from patients admitted to ICUs.

A total of 2308 patients were admitted to ICUs in the study period, from which 3056 samples were taken and sent to the microbiology lab for the bacterial culture. The infection rate among ICU patients was found to be 53.40%.

The demographic variables of culture-positive patients in this study revealed that the number of males and females developing infection inside the ICU was almost equal. In many studies, the infection rate in men was found to be higher than the one in women [11, 12].

The majority of the isolates were from 1 to 11 years old children followed by adults from 18 to 45 years of age (28.03%). Bloodstream infections (BSIs) accounted for the most common infection in the ICU setting (39.08%) followed by respiratory infections (29.45%). This finding is similar to that of the study performed by Fahim in 2021 in Egypt, where the highest number of pathogens was isolated from blood cultures (44.84%), followed by urine (41.41%), and then wound swabs (13.75%) [13]. However, studies performed by Satyajeet et al. in 2016 and Moolchandani et al. in 2017 in different parts of India showed that pneumonia was the most common ICU infection [12, 14].

Nonfermenting Gram-negative bacilli (NF-GNB) have emerged as important hospital-acquired pathogens because of an increasingly unreasonable and irrational use of broad-spectrum antimicrobials. Usually, these pathogens are inhabitants of nature, particularly in soil and water. In the hospital environment, they may be isolated from instruments such as ventilators, tubing, and from the skin of healthcare workers [12]. Also, in the present study, *Acinetobacter* sp. (33.02%) was the most common organism isolated from ICUs. This finding is in concordance with the study conducted by Mehta et al. in 2015 in Ahmedabad, where *Acinetobacter* sp. (30.92%) was the commonest organism [1]. Next to *Acinetobacter* spp., Enterobacteriaceae GNB such as *Klebsiella* sp. (20.89%) were the second most common organism in this study. In a study from the Dominican Republic in 2020, *E.coli* represented 17.7% of the total isolated microorganisms from ICUs, *Pseudomonas* sp. and *Acinetobacter* sp. represented 12.6% and 8.0% of the total, respectively, while *S. aureus* accounted only for 10% [15].

Other NF-GNB isolated less frequently in this study include *Salmonella Typhi, Proteus* sp. (0.30%), *Morganella morganii* (0.24%), *Burkholderia cepacia* (0.06%), *Stenotrophomonas* sp. (0.06%), and *Sphingomonas* sp. (0.06%).

NF-GNB were also the most common organism in the study conducted by Moolchandani et al. in 2017 in Puducherry; however, the predominating organisms were *Pseudomonas* sp. (19.1%) followed by *Acinetobacter* spp. (17.5%) [12]. In Asian countries like India, *Pseudomonas* spp. have been the most common organism isolated from ICUs [4, 5, 12, 16].

Gram-positive cocci such as *Staphylococcus aureus* (16.78%) and *Enterococcus* spp. (3.73%) were also seen to cause infections in the ICU settings in this study. The global scenario shows that Gram-positive organisms are more common in the western world (North America and Europe) than in Asian countries [17–19].

Finally, the resistance patterns of various microorganisms were analyzed in this study. More than 80% of *Acinetobacter* spp. were found to be resistant to third-generation cephalosporins, aminoglycosides, and carbapenems. Minocycline (56.31% S) and colistin (100% S) were the most effective drugs for *Acinetobacter* sp. The results were similar to those of the study performed by Said et al. in 2021 in Saudi Arabia, where *A. baumannii* was found to be the most resistant pathogen isolated from clinical specimens and the isolates were fully resistant to almost all antibiotics tested, except for amikacin (61.25%), colistin (5%), and ertapenem (0%) [11]. Given the noteworthy prevalence of *Acinetobacter species*, empirical therapy in the ICU setting may need to include agents effective against this organism depending on local resistance patterns. Consideration may be given to newer combination agents such as imipenem-cilastatin, ceftazidime-avibactam, or ceftolozane-tazobactam. Colistin in combination with carbapenem would be a potential option for the management of such drug-resistant bugs [20].

A wide range of antibiotics were ineffective in the treatment of Enterobacteriaceae GNB. *E. coli* was highly resistant to *β*-lactam antibiotics including 3rd generation cephalosporins and fluoroquinolones. Least resistance was recorded in chloramphenicol (33.3%), amikacin (40.74%), and gentamicin (42.14%). *Klebsiella* sp. was found to be more resistant than *E.coli*, where least resistance was observed in tetracycline (43.97%) and doxycycline (55.84%). Colistin was again found to be 100% susceptible to both organisms. For this reason, it is imperative to reserve colistin until antimicrobial susceptibility patterns mandate its use. Similar results were seen in a study conducted by Fahim in 2021 in Egypt, where Gram-negative isolates showed the least frequency of resistance against nitrofurantoin (52.5%), amikacin (58.01%), followed by imipenem (59.78%), and meropenem (61.82%) [13].

*P. aeruginosa* was found to be moderately resistant to anti-pseudomonal cephalosporins (41.5%) and carbapenems (55.2%). Least resistant drugs included piperacillin-tazobactam (29.89%), aztreonam (21.82%), and colistin (0%). Moolchandani et al. had reported a similar resistance pattern of *Pseudomonas* sp. to various classes of drugs ranging from 25 to 70% [12]. In many studies, *P. aeruginosa* has been recorded as one of the organisms showing high levels of resistance (over 80% R) to routine drugs used for treatment, including carbapenems [11].

In this study, the production of extended-spectrum *β*-lactamases (ESBLs) was seen to be 16.0% in *E. coli* and *Klebsiella* sp., whereas only 4.10% was seen in *Acinetobacter* sp. Chakraverti in 2015 in Bihar reported a higher rate of ESBL production (30–50%) [5]. While the prevalence of ESBL-producing isolates was reported extremely high (67–84%) in a study conducted by Uc-Cachon in Mexico in 2019 [21].

The analysis of the antibiotic susceptibility profile of *S. aureus* revealed that 82.78% strains were methicillin-resistant (MRSA). MRSA has been reported to vary from 40 to 57% in various studies [12, 15]. Methicillin resistance was observed to be higher in coagulase-negative *Staphylococcus* spp. (CONS) accounting for 82.05%. This result is frightening as this might lead to an increase in use of reserve antibiotics such as vancomycin, which increases the possibility of development of vancomycin resistance. However, *S. aureus* was found to be totally susceptible to vancomycin in this study. Similar results were seen in a study conducted by Faim in 2021 in Egypt, where *S. aureus* exhibited high resistance rates to many antibiotics including penicillin (97.1%), gentamicin (73.91%), and all beta-lactams. Also, CONS showed comparable *β*-lactam resistance rates to *S. aureus* with a slightly higher level of methicillin resistance (77.6%), as well as 100% susceptibility to linezolid and vancomycin [13].

In this study, *Enterococci* spp. expressed a high level of resistance to erythromycin (81.97%), ciprofloxacin (79.63%), tetracycline (74.58%), penicillin (74.51%), and high-level gentamicin (65.57%). This pattern of resistance obviates the synergistic action of *β*-lactam and aminoglycoside agents. Also, the level of VRE was seen to be 16.67% of total *Enterococci* spp. isolates. In a study conducted by Pawar et al. in 2016 in Maharashtra, VRE was reported in 12% of isolates [14]. VRE has been reported to be as high as 63% by Despotovic et al. in 2020 [22]. In developed countries, such as in Europe, the proportions of vancomycin-resistant *E. faecium* were reported to increase from 8.1% in 2012 to 19.0% in 2018 [23]. The limitations of the study included a lack of adequate data on clinical information and an inability to analyze the rate of coresistance among different pathogens. Also, the treatment choices and outcomes of the patients with infections could not be analyzed as it was a retrospective study.

Clinicians could use this evidence-based knowledge directly to tailor antibiotic regimens to the specific pathogens identified, optimizing the chances of effective treatment [24]. The findings can be incorporated into local antibiotic guidelines and protocols for the ICU. The study results may stimulate further research into new treatment modalities or preventive measures for infections caused by these prevalent organisms. The study encourages regular surveillance within the ICU and broader healthcare environments to track changes in resistance patterns, allowing for timely adjustments in empirical therapy and infection prevention strategies.

## 5. Conclusion

Bacteriological profiles and antimicrobial susceptibility data are important to identify emerging drug-resistant pathogens, provide opportunities for new drug development, and form local antimicrobial policy, which further helps in having national data. Our study has shown 100% colistin susceptibility for *E. coli, Acinetobacter* sp., *Klebsiella pneumoniae*, and *Staphylococcus aureus*; therefore, it should be considered as the most effective drugs. Evidence-based knowledge regarding the local bacterial organisms and their antimicrobial resistance pattern is pivotal in deciding empirical drug therapy, ultimately leading to the management of antimicrobial resistance.

The Global Antibiotic Resistance Partnership (GARP) guidelines recommend a multipronged strategy in low- and middle-income countries to optimize the use of antibiotics and reduce antibiotic resistance. The priority actions recommended national surveillance of antibiotic use and antibiotic resistance, as well as strengthening of infection control committees in hospitals. Hence, studies like this are particularly important in countries like India, where infection control practices and antimicrobial policies need to be strengthened to boost antibiotic stewardship and help in the reduction of antibiotic resistance and patient morbidity and mortality in the long run.

## Figures and Tables

**Table 1:** Clinical specimen distribution and demographic profile of culture-positive patients (*n* = 1632).

	Number	Percentage of positivity (%)
Gender		
Male	830	50.86
Female	802	49.14
Age group		
0-1 year	207	12.71
>1–11 years	510	31.21
>11–18 years	132	8.10
>18–45 years	457	28.03
>45 years	326	19.95
Distribution among ICUs		
MICU	504	30.88
SICU	450	27.57
PICU	678	41.55
Sample type		
Blood	650	39.08
Respiratory samples (BAL, tracheal aspirate, sputum, ET tube, nasal swabs)	480	29.45
Exudates (pus, wound swabs, stitch line swabs, liver abscess)	119	7.32
Body fluids (peritoneal, pleural, pericardial fluids)	109	6.71
Urine	99	6.10
CSF	22	1.35
HVS	23	1.40
Tissue	08	0.45
Others (oral swabs, catheter tips, GA, throat swab, bile)	61	3.75
Not mentioned	61	3.75

*Note.* ICUs = intensive care units, MICU = medical ICU, SICU= surgical ICU, PICU = paediatric ICU, BAL = bronchoalveolar lavage, CSF = cerebrospinal fluid, HVS = high vaginal swab, ET = endotracheal, and GA = gastric lavage.

**Table 2:** Distribution of bacterial isolates in culture-positive patients (*n* = 1632).

Organisms	Number	Percentage (%)
*Acinetobacter* sp.	539	33.02
*Klebsiella pneumoniae ss. pneumoniae*	341	20.89
*Staphylococcus aureus ss. aureus*	274	16.78
*Escherichia coli*	226	13.8
*Pseudomonas aeruginosa*	118	7.23
*Enterococcus* spp.	61	3.73
*Staphylococcus, coagulase negative*	39	2.38
*Salmonella typhi*	7	0.42
*Klebsiella oxytoca*	6	0.36
*Proteus mirabilis*	4	0.24
*Morganella morganii ss. morganii*	4	0.24
*Citrobacter* spp.	2	0.12
*Aeromonas* spp.	1	0.06
*Burkholderia cepacia*	1	0.06
*Burkholderia* spp.	1	0.06
*Enterobacter* spp.	1	0.06
*Proteus vulgaris*	1	0.06
*Providencia stuartii*	1	0.06
*Salmonella Paratyphi* A	1	0.06
*Sphingomonas* sp.	1	0.06
*Stenotrophomonas maltophilia*	1	0.06
*Streptococcus pyogenes*	1	0.06
*Vibrio vulnificus*	1	0.06
Total	1632	

^*∗*^ *sp*: species.

**Table 3:** Antimicrobial susceptibility pattern of predominant Gram-positive cocci.

Antibiotic name	*Staphylococcus aureus*			*Enterococcus* sp		
	*R* (%)	*I* (%)	*S* (%)	*R* (%)	*I* (%)	*S* (%)
Ampicillin	—	—	—	74.51	0.00	25.49
Penicillin G	96.34	0.00	3.66	—	—	—
Amoxicillin/clavulanic acid	—	—	—	71.43	2.38	26.19
Gentamicin-high level	—	—	—	65.57	0.00	34.43
Gentamicin	21.69	9.93	63.38	—	—	—
Ciprofloxacin	58.30	12.11	29.60	79.63	7.41	12.96
Norfloxacin	—	—	—	93.75	0.00	6.25
Erythromycin	77.01	7.66	15.33	81.97	13.11	4.92
Nitrofurantoin	—	—	—	42.86	9.52	47.62
Linezolid	0.00	0.00	100.00	0.00	0.00	100.00
Vancomycin	0.00	0.00	100.00	16.67	0.00	83.33
Teicoplanin	0.00	0.00	100.00	23.73	1.69	74.58
Chloramphenicol	18.08	2.26	79.66	14.29	0.00	85.71
Doxycycline	8.42	5.86	85.71	58.62	5.17	36.21
Minocycline	3.62	2.90	93.48	69.23	3.85	26.92
Tetracycline	19.59	5.86	74.55	74.58	5.17	19.98
Cefoxitin (MRSA)	82.78	0.00	17.22	—	—	—

## Data Availability

The data used to support the findings of this study are included within the article.
